# Chiral Bifunctional Thioureas and Squaramides Grafted into Old Polymers of Intrinsic Microporosity for Novel Applications

**DOI:** 10.3390/polym11010013

**Published:** 2018-12-21

**Authors:** María Valle, Laura Martín, Alicia Maestro, José M. Andrés, Rafael Pedrosa

**Affiliations:** Instituto CINQUIMA and Departamento de Química Orgánica, Facultad de Ciencias, Universidad de Valladolid, Paseo de Belén 7, 47011 Valladolid, Spain; mariavallealvarez@hotmail.com (M.V.); laura.martinm@uva.es (L.M.); amaestro@qo.uva.es (A.M.)

**Keywords:** organocatalysis, supported catalysts, polymers of intrinsic microporosity, asymmetric nitro-Michael reaction, thioureas, squaramides

## Abstract

We have prepared different polymeric chiral bifunctional thioureas and squaramides by modification of the very well-known polymers of intrinsic microporosity (PIM), specifically PIM-1 and PIM-CO-1, to be used as recoverable organocatalysts. The installation of the chiral structures into the polymers has been done in two or three steps in high yields. The catalytic activity of the resulting materials has been proved in the stereoselective nitro-Michael addition and in a cascade process, which allows the synthesis of enantioenriched 4H-chromene derivatives. Squaramide **II** and thiourea **III** have been used in six cycles maintaining their activity.

## 1. Introduction

Since the discovery of polymers of intrinsic microporosity (PIMs) fifteen years ago [1], a great deal of work has been developed around their applications in physicochemical processes [2,3,4]. PIMs are characterized by their lack of rotational freedom along the polymeric chain, which confer a lot of void space, and consequently, interconnected pores along their structures.

The solubility in different organic solvents makes PIMs more advantageous than inorganic porous materials in terms of processability, allowing the formation of membranes with different applications. In that way, PIMs have been extensively studied in gas separation [5,6,7,8,9,10,11], nanofiltration and phase separation [12,13,14,15], hydrogen storage [16,17,18,19], and catalysis for different transformations [3,13,20].

The most classical way to obtain PIMs employs the co-polymerization of bis-catechol derivatives with perhalobenzenes activated with electron withdrawing groups to form a dibenzodioxin linkage [21]. Interestingly, only the archetypal PIM-1 [12] has been subjected to additional modifications based on the transformation of the nitrile groups. This group has been converted into carboxylic acids [22,23], thioamides [24], amidoximes [25], tetrazoles [26,27], and amides [28] and has been reduced to diamines [29], but all these transformations have been directed to the synthesis of modified PIM-1 to get improvement in gas permeation and in diffusion processes. On the other hand, some other types of polymers, such as chiral helical polymers [30] or copolymers [31] with different pendant substituents [32] have been successfully used as catalysts in enantioselective Rauhut-Currier [33] or aldol [34] reactions.

Our interest in the preparation of recoverable and reusable catalysts that are able to promote enantioselective reactions [35,36,37,38] led us to consider the synthesis of PIMs decorated with chiral thioureas and squaramides and to study their ability to act as organocatalysts in these kinds of transformations. 

We selected the most studied PIM-1 as the starting material for the direct synthesis of thioureas derived from *L*-valine or cyclohexanediamine, and *L*-valine-derived squaramide. As an alternative, PIM-CO-100 [39], which differs from PIM-1 in the nature of the contortion unit, was selected for the synthesis of two different thioureas and one squaramide. We report here the synthesis of these materials and their use as organocatalysts in some of the most commonly studied stereoselective transformations.

## 2. Materials and Methods

### 2.1. Materials

Commercially available reagents were used as purchased without further treatment. Solvents were dried and stored over microwave-activated 4 Å molecular sieves. 5,5′,6,6′-Tetrahydroxy-3,3,3′,3′-tetramethyl-1,1′-spirobisindane was purchased from Alfa Aesar (Karlsruhe, Germany), and 2,3,5,6-tetrafluorophtalonitrile was from Sigma-Aldrich (Madrid, Spain. 1-((1R, 2R)-2-Aminocyclohexyl)piperidine [40], 9,10-Dimethyl-9,10-ethano-9,10-dihydro-2,3,6,7-tetrahydroxy-anthracene [41], isothiocyanates **1** and **4** [42], and chiral hemisquarate **2** [43] were prepared as described in the literature. 1,3-Dicarbonyl compounds **5a**–**c** and *trans*-β-nitrostyrene **6** are commercially available, and they were used as received. 

### 2.2. Characterization Techniques

Elemental analysis of the starting PIMs, their corresponding modifications, and final catalysts were determined using a LECO CHNS-932 at the Elemental Analysis Center of the Complutense University of Madrid (Madrid, Spain). Infrared spectra were carried out using a Perkin-Elmer spectrum One FT-IR spectrometer (PerkinElmer, Madrid, Spain and spectra was reported in frequency of absorption (only the structurally most important peaks are given). Microstructural morphology of the catalysts was observed by environmental scanning electron microscopy (ESEM) in low vacuum mode (FEI Quanta 200 FEG) at the UM-PCUVa (Valladolid Spain). Samples for atomic force microscopy (AFM) were prepared on mica by placing a solution of the catalyst in *N*,*N*-dimethylformamide (DMF). AFM was performed in air at 25 °C using a MultiMode 8 AFM attached to a Nanoscope V electronics (Veeco Instruments, Santa Barbara, CA, USA) in tapping-mode at the Scientific and Technological Center of the University of Barcelona (Spain). Both topography and phase signals images were recorded with 512 × 512 data points. D-line specific rotations ([α]_D_^25^) were measured in CHCl_3_ (10 mg/mL) at 25 °C using a Perkin-Elmer 341 digital polarimeter (PerkinElmer, Madrid, Spain. ^1^H NMR spectra were recorded on Bruker equipment at 400 or 500 MHz, and ^13^C NMR were recorded at 100 MHz in CDCl_3_ as the solvent. Chemical shifts are reported in ppm relative to tetramethylsilane (TMS). Data are reported as follows: Chemical shift, multiplicity (*s* = singlet, *d* = doublet, *t* = triplet, *q* = quartet, *m* = multiplet, *br* = broad), coupling constants in Hertz, and integration. Flash chromatography was carried out using silica gel (230–240 mesh). Thin layer chromatography TLC analysis was performed on glass-backed plates coated with silica gel 60 and an F254 indicator and was visualized by either UV irradiation or by staining with phosphomolybdic acid or potassium permanganate solutions. Chemical yields refer to pure isolated substances. High-resolution mass spectrometry analysis (HRMS) was performed by a quadrupole spectrometer with Time-of-Flight TOF analyzer by the Technical-Scientific Service at the University of Valladolid (Valladolid Spain). Chiral high performance liquid chromatography HPLC analysis was performed on a JASCO HPLC system (JASCO PU-2089 pump and UV-2075 UV/Vis detector) and on a Hewlett-Packard 1090 Series II instrument equipped with a quaternary pump using Daicel Chiralcel OD (250 × 4.6 mm), Chiralpak AD-H (250 × 4.6 mm), and Phenomenex Lux Amylose-1 (250 × 4.6 mm). Thermogravimetric analysis (TGA) was performed under nitrogen, using a Mettler, Mod. TGA/SDTA 851e with a heating rate of 10 °C min^−1^ over a temperature range of 50–850 °C. 

### 2.3. Synthesis of Grafted PIMs (I-VI)

**Scheme 1:**
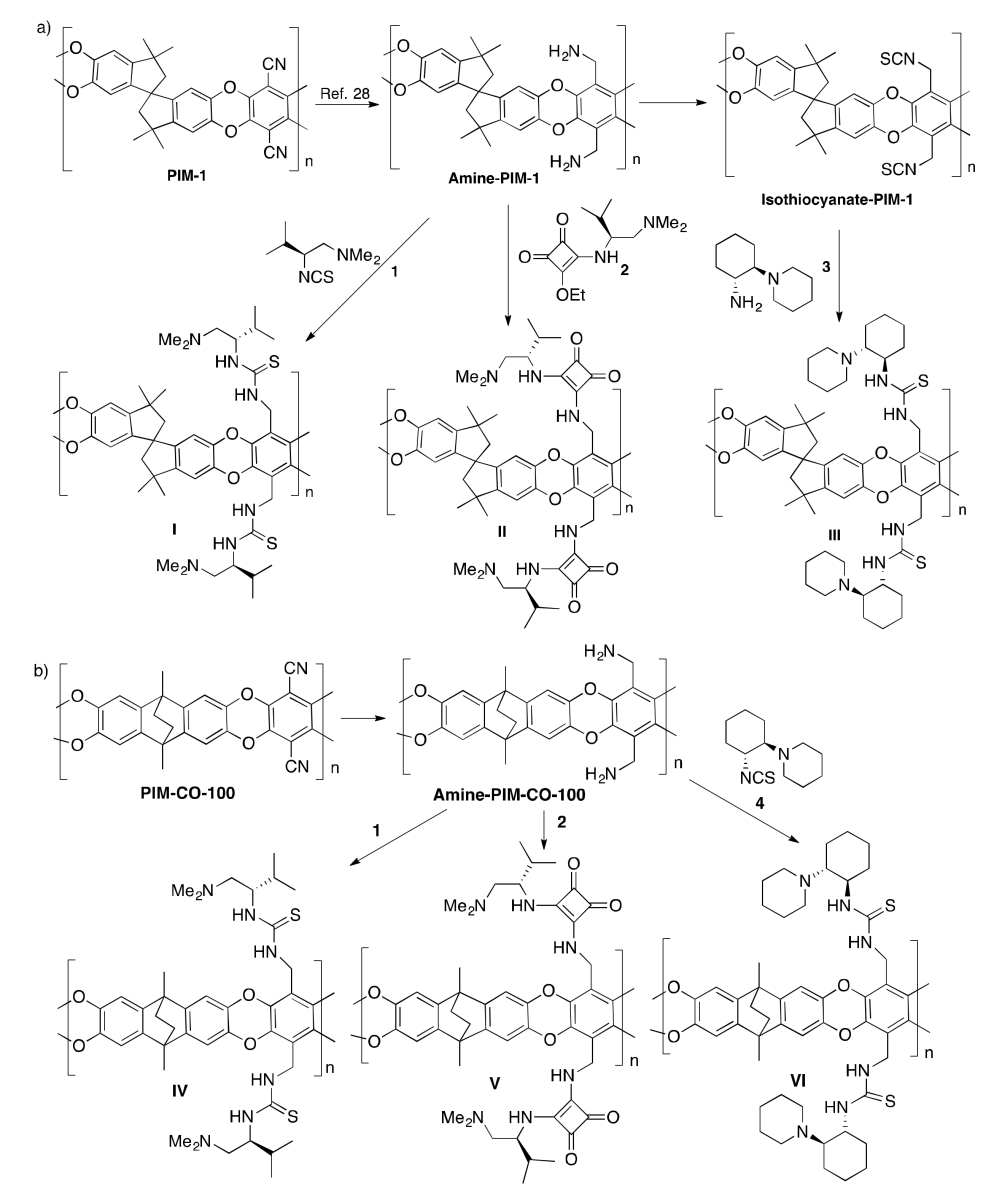
(**a**) Synthesis of thioureas **I**, **III**, and squaramide **II** from PIM-1; (**b**) Thioureas (**IV**, **VI**) and squaramide **V** prepared from PIM-CO-100.

We synthesized two different substituted bifunctional chiral thioureas and one squaramide into the backbone of both PIM-1 and PIM-CO-100 as summarized in Scheme 1. PIM-1, prepared from 5,5′,6,6′-tetrahydroxy-3,3,3′,3′-tetramethyl-1,1′-spirobisindane and 1,4-dicyanotetraflurobenzene, was reduced with diborane to the well-known amine-PIM-1 [29]. PIM-CO-100 was obtained, as previously described [44], by condensation of 9,10-Dimethyl-9,10-ethano-9,10-dihydro-2,3,6,7-tetrahydroxy-anthracene [41] with 1,4-dicyanotetrafluorobenzene and was reduced to amine-PIM-CO-100 with borane-dimethylsulfide complex. These amines were transformed into thioureas or squaramide in two different ways.

#### 2.3.1. Synthesis of Valine-Derived Thioureas (**I**) and (**IV**)

These polymers were obtained by reaction of isothiocyanate **1** with amine-PIM-1 (Scheme 1a) or amine-PIM-CO-100 (Scheme 1b) respectively. A suspension of amine-PIM-1 (150 g, 0.48 mmol, 1 equiv.) in anhydrous CH_2_Cl_2_ was reacted with a solution of isothiocyanate **1** (143 mg, 0.83 mmol, 2 equiv.) in CH_2_Cl_2_ under nitrogen atmosphere for 24 h at room temperature. The dark brown solid was collected by filtration and thoroughly washed with CH_2_Cl_2_, acetone, and dried under vacuum to give 187 mg of grafted-thiourea polymer **I** as a dark brown solid (81% yield). An effective functionalization, *f* = 1.54 mmol g^−1^, was calculated on the basis of sulfur elemental analysis (C: 53.17; H: 5.56; N: 7.05; S: 4.94). Polymeric thiourea **IV** was obtained in the same way starting from amine-PIM-CO-100 (150 mg, 0.56 mmol, 1 equiv) and isothiocyanate **1** (143 mg, 0.83 mmol, 2 equiv), obtaining 195 mg of polymer **IV** (80% yield). An effective functionalization, *f* = 1.22 mmol g^−1^, was calculated on the basis of sulfur elemental analysis (C: 61.30; H: 6.12; N: 7.32; S: 3.90).

#### 2.3.2. Synthesis of Valine-Derived Squaramides (**II**) and (**V**)

A suspension of amine-PIM-1 (150 mg, 0.48 mmol) in anhydrous CH_2_Cl_2_ (10 mL) was added to a solution of hemisquarate **2** (122 mg, 0.96 mmol, 2 equiv.) at 0 °C under nitrogen atmosphere. The resulting suspension was stirred for three days at room temperature. The material was collected by filtration, washed with CH_2_Cl_2_ and MeOH, and dried under vacuum at 60 °C to give 193 mg of squaramide-PIM-1 (**II**) (92% yield) (Scheme 1a). An effective functionalization, *f* = 1.44 mmol g^−1^, was calculated on the basis of nitrogen elemental analysis (C: 59.33; H: 5.54; N: 6.06). Squaramide **V** was obtained from amine-PIM-CO-100 (150 mg, 0.56 mmol) by reaction with hemisquarate **2** (285 mg, 1.12 mmol, 2 equiv.) as described above. After filtration, washing, and drying, 198 mg of **V** was obtained (76% yield) (Scheme 1b). An effective functionalization, *f* = 1.52 mmol g^−1^, was calculated on the basis of nitrogen elemental analysis (C: 60.94; H: 5.88; N: 6.40).

#### 2.3.3. Synthesis of Cyclohexanediamine-Derived Thioureas (**III**) and (**VI**)

Thiourea **III** was prepared in two steps starting from amine-PIM-1. Isothiocyanate PIM-1 was obtained by a modified procedure previously published [45]. A suspension of amine-PIM-1 (150 mg, 0.48 mmol, 1 equiv.) in dichloromethane (10 mL) was allowed to swell for 30 min. Then, triethylamine (0.27 mL, 1.92 mmol, 4.0 equiv.) and thiophosgene (44 µL, 0.58 mmol, 1.2 equiv.) were added dropwise at room temperature After shaking for 3 h, the reaction mixture was filtered and the material was washed with dichloromethane (30 mL), tetrahydrofurane (30 mL), and dichloromethane (30 mL) and dried over reduced pressure at 60 °C for 12 h to yield 163 mg of isothiocyanate-PIM-1 (0.29 mmol, 96%). An effective functionalization, *f* = 1.78 mmol g^−1^, was calculated on the basis of sulfur elemental analysis (C: 52.94; H: 4.33; N: 4.12; S: 5.68). 

Isothiocyanate-PIM-1 (150 mg, 0.27 mmol, 1 equiv.) was swollen for 30 min in anhydrous CH_2_Cl_2_ (10 mL), and 1-((1*R*,2*R*)-2-aminociclohexyl)piperidine **3** (97 mg, 0.53 mmol, 2 equiv.) was added at 0 °C under nitrogen atmosphere. The reaction mixture was allowed to warm slowly to room temperature over a period of 3 h and then was stirred for a further 12 h at room temperature. The reaction mixture was filtered, and the polymer was washed with CH_2_Cl_2_ (30 mL), MeOH (30 mL), and CH_2_Cl_2_ (30 mL), and finally, the dark brown solid was dried under vacuum to produce 185 mg of thiourea (**III**) (0.36 mmol, 93%), (Scheme 1a). An effective functionalization, *f* = 1.36 mmol g^−1^, was calculated on the basis of sulfur elemental analysis (C: 58.41; H: 5.55; N: 6.45; S: 4.34). Thiourea **VI** was obtained (Scheme 1b) by direct condensation of amine-PIM-CO-100 (150 mg, 0.56 mmol, 1 equiv.) with 1-((1R,2R)-2-isothiocyanatocyclohexyl)piperidine **4** (251 mg, 1.12 mmol, 2 equiv) as previously described [42] to produce 211 mg of polymer (77% yield). An effective functionalization, *f* = 1.36 mmol g^−1^, was calculated on the basis of sulfur elemental analysis (C: 61.93; H: 6.33; N: 7.40; S: 4.36).

### 2.4. General Procedure for Asymmetric Reactions

#### 2.4.1. General Procedure for Stereoselective Nitro-Michael Addition

1,3-Dicarbonyl compound **5a**–**c** (0.6 mmol, 2.0 equiv.) was added to a mixture of *trans*-β-nitrostyrene **6** (0.3 mmol) and the corresponding catalyst (**I**–**VI**) (5 mol %, calculated on the basis of its effective functionalization given in mmol g^−1^). The reaction was stirred at room temperature in a Wheaton vial until consumption of the starting material (monitored by TLC). The catalyst was filtered off and washed with dichlorometane (3 × 0.5 mL). The solvent was removed from the filtrate under reduced pressure, and the residue was purified by flash chromatography on silica gel with hexane/EtOAc (from 20:1 to 8:1) as the eluent to afford the corresponding pure Michael adduct **7a**–**c**.

Racemic reference samples were prepared using 1,4-diazabicyclo[2.2.2]octane (DABCO) as a base in the same conditions as that for the asymmetric reaction.

#### 2.4.2. General Procedure for Preparation of 2-Amino-4-(nitromethyl)-4H-chromene-3-carbonitrile from 2-(2-Nitrovinyl)phenol Derivative

A mixture of malononitrile **9** (0.6 mmol, 2.0 equiv.) and the grafted PIM’s catalyst (5 mol%, calculated on the basis of its effective functionalization given in mmol g^−1^) in dichloromethane (0.5 mL) was added to 2-(2-nitrovinyl)phenol **8** (0.3 mmol). The mixture was stirred at room temperature in Wheaton vial until consumption of the starting material (monitored by TLC). The catalyst was filtered off and washed with dichloromethane (3 × 0.5 mL) and methanol (3 × 0.5 mL). After removal of the solvent under reduced pressure, the crude mixture was purified by silica gel flash column with hexane/EtOAc (3:1) as the eluent to produce the corresponding pure product **10**.

### 2.5. Recyclability of the Grafted PIM’s Catalysts in the Asymmetric Reactions

The catalysts **II** and **III**, used in the recyclability studies were recovered by filtration when the reaction was finished, washed with dichloromethane and methanol, and dried under vacuum until a constant weight. These materials were used directly for the next cycle.

## 3. Results and Discussion

### 3.1. Polymerization, Post-Modification, and Structural Characterization of PIMs

**Figure 1:**
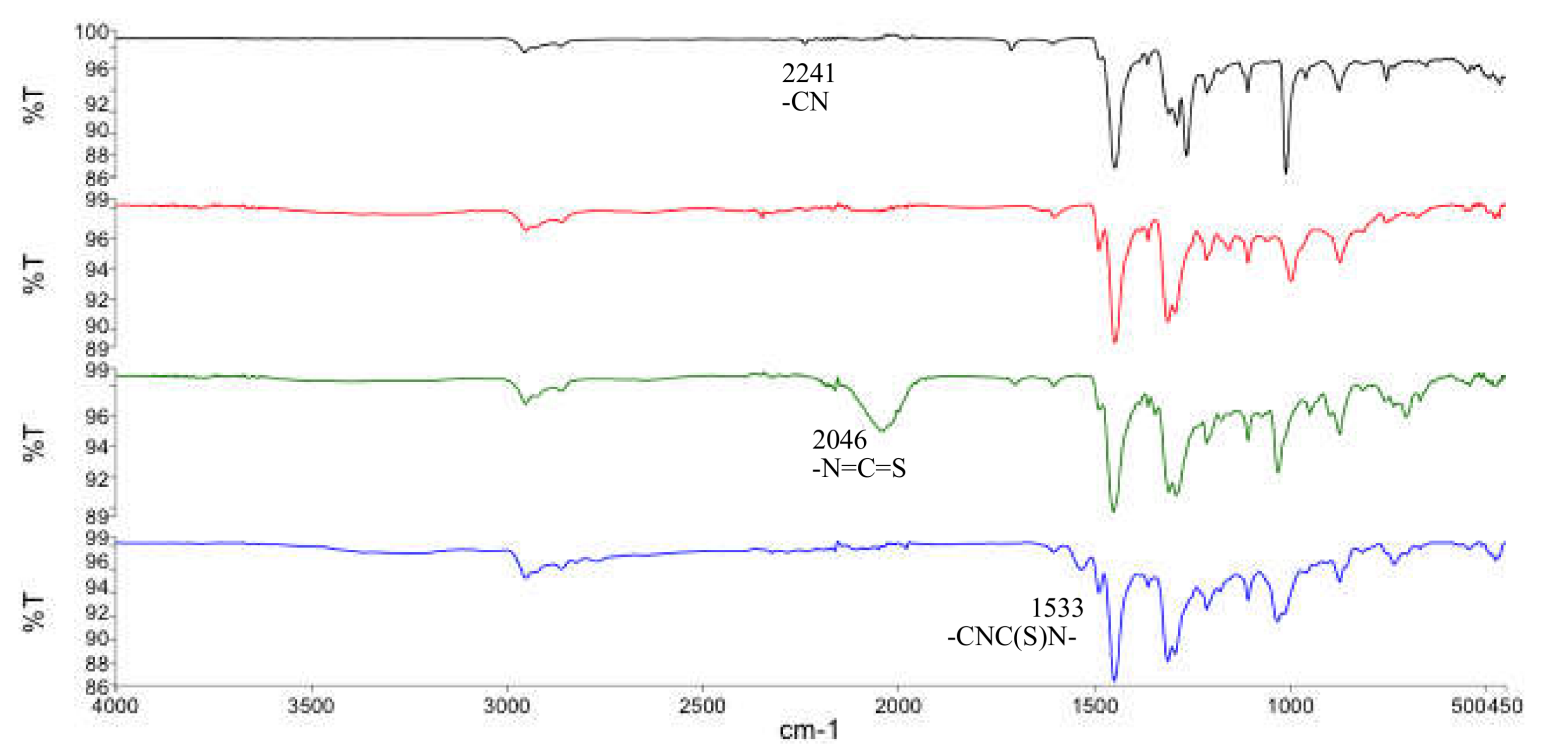
Fourier transform infrared spectroscopy (FTIR) spectra of PIM-1, Amine-PIM-1, isothiocyanate-PIM-1, and catalyst **I**.

The synthesis and post-modifications of well-defined PIMs, subsequent Amine-PIMs, and grafted PIM’s catalysts were monitored by infrared spectroscopy. As an example, Figure 1 compares IR spectra of the PIM-1 (black), amine-PIM-1 (red), isothiocyanate-PIM-1 (green), and catalyst **I** (blue). It is clear that all the CN groups in starting PIM-1 have been reduced to amine groups because the absorption at 2241 cm^−1^ is not present, and a characteristic broad band of amine at 3200–3500 cm^−1^ has appeared. The peak at 2046 cm^−1^ corresponds to the isothiocyanate group in the isothiocyanate-PIM-1 polymer, and the stretching vibration of C–N at 1533 cm^−1^ confirms the presence of the thiourea substituents in **I**.

^1^H NMRand ^13^C NMR spectroscopy were employed to confirm the chemical structure of the soluble the starting PIMs in CDCl_3_ (see Supplementary Materials, and the results of the elemental analysis of the starting and modified PIMs indicated that the transformations occurred in high yields.

In addition, the thermal behavior in relation to the chemical modifications of the PIM-1 that produced the final grafted catalysts **I**–**III** was investigated with TGA.

**Figure 2:**
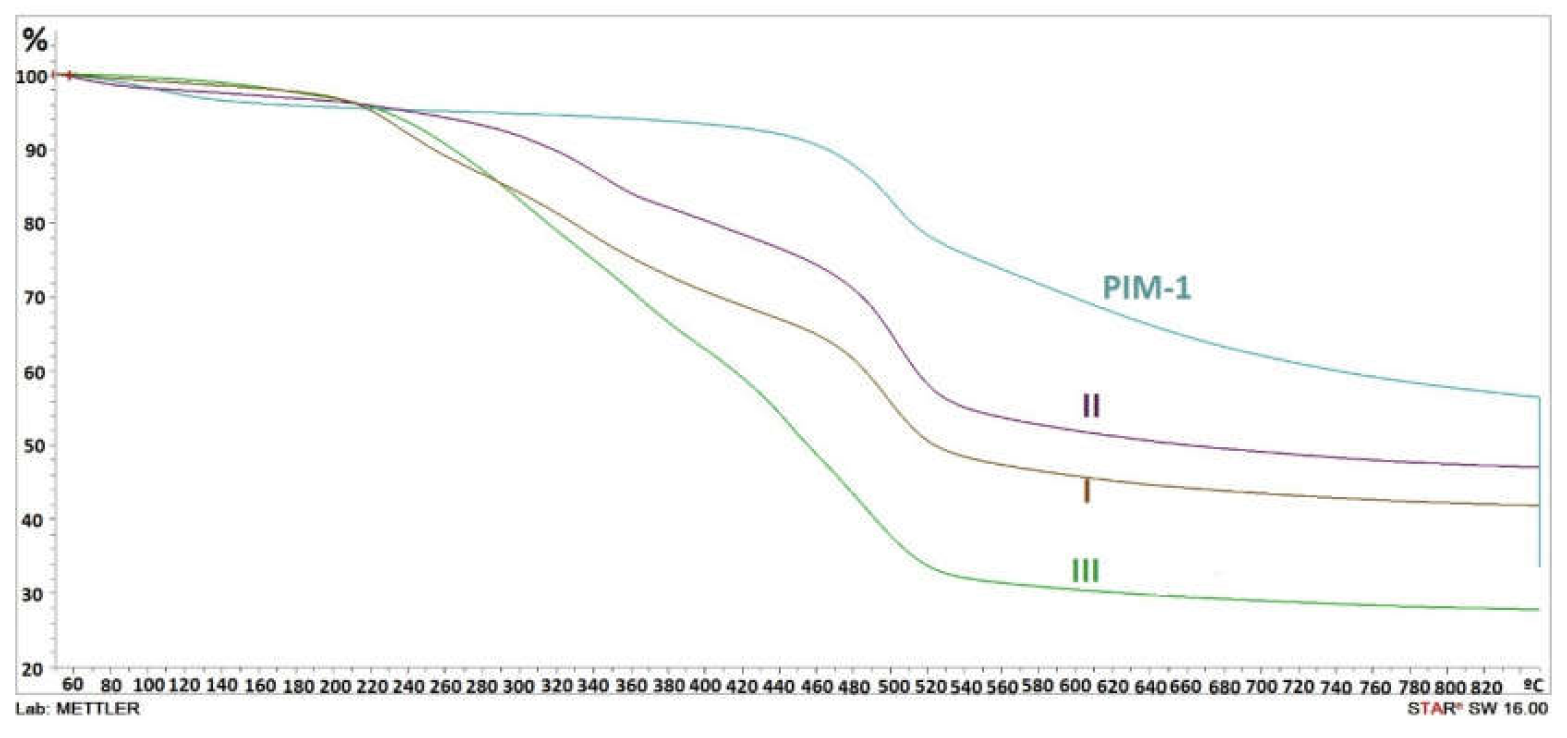
Thermogravimetric analysis (TGA) thermograms comparison of PIM-1 and catalysts **I**–**III** under nitrogen atmosphere.

Figure 2 shows TGA thermograms. It is well-known that PIM-1 powder polymer have an extraordinarily high thermal stability of above 400 °C with a single-step decomposition pattern [28,29]. However, the nitrile replacement with thiourea or squaramide groups resulted in a decrease in the thermal stability. PIM-1 and the catalysts **I**–**III** gradually conveyed approximately until 4% of weight loss at low temperatures between 50 and 150 °C was attributed to the loss of volatiles, and as temperature was ramped up to 300 °C for catalyst **II** and up to 220 °C for thioureas **I**–**III**, the weight loss became more dramatic due to the decomposition of the thiourea and squaramide groups amongst other thermal degradation processes that could also occur at temperatures ramped up to 450 °C.

**Figure 3:**
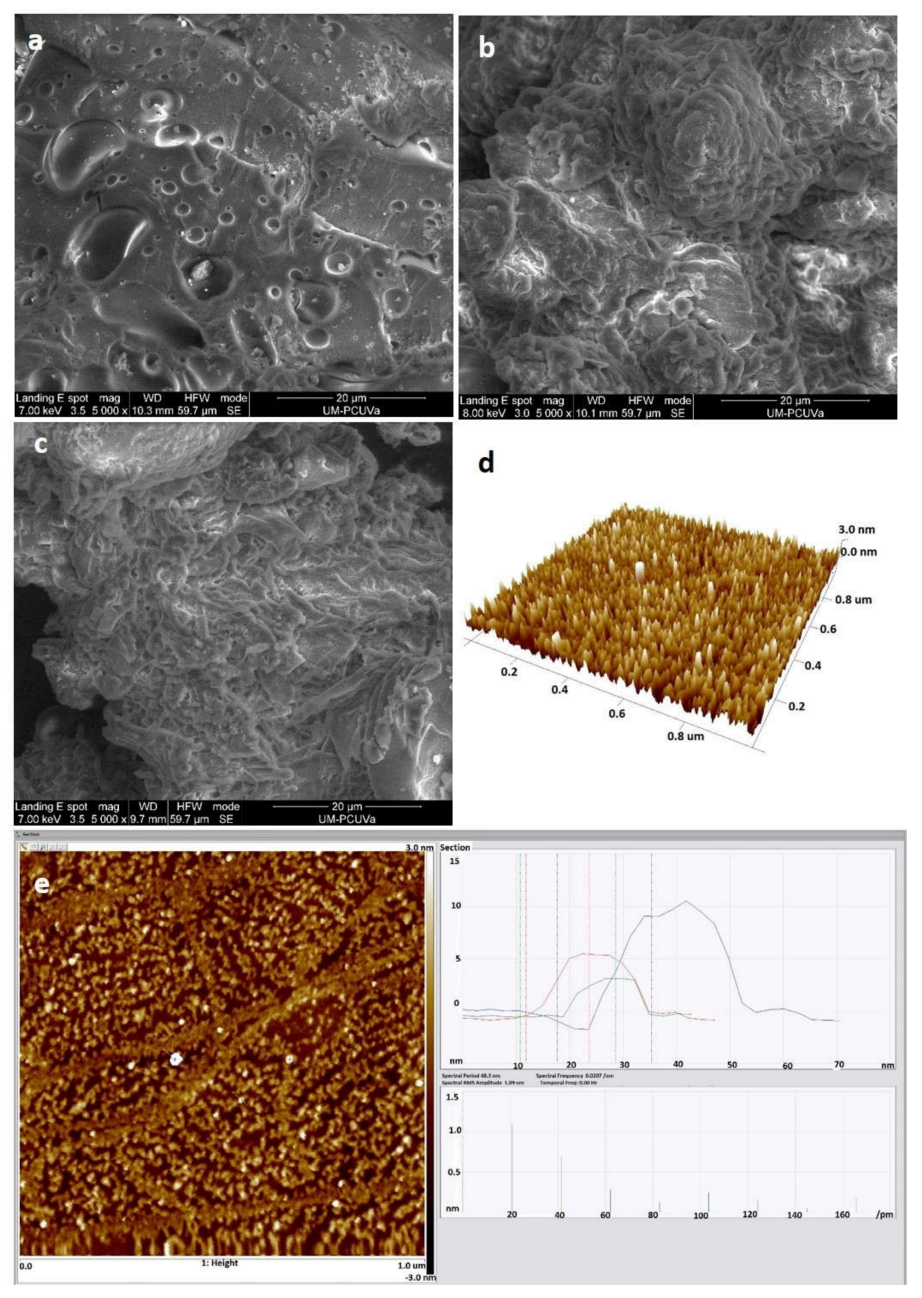
Environmental scanning electron microscopy (ESEM) micrographs of PIM-1 (**a**), Amine-PIM-1 (**b**), and catalyst **III** (**c**). Tapping-mode atomic force microscopy (AFM) section and 3D images of catalyst **III** (**d**,**e**).

Furthermore, the microstructural morphology of the polymers was observed by ESEM. As an example, some micrographs of the starting PIM-1, Amine-PIM-1, and catalyst **III** are showed in Figure 3. Inspection of these images reveals the intrinsic porosity of the materials and the observation that subsequent modifications such as dissolution and swelling in different solvents, changes in the chemical composition, and resulting alterations in surface energy, amongst others, might contribute to enhancing the surface roughness. These changes can be useful in improving the catalytic activity of the grafted PIM’s catalysts. In light of these results, AFM was used to observe the morphology of the catalyst **III**. The section image shows pore size between 15 and 40 nm (Figure 3e, right), and additionally, the 3D representation clearly shows microporous structure, indicating that the modification of the starting PIMs does not disturb their characteristic intrinsic microporosity.

### 3.2. Evaluation of Catalytic Activity

**Scheme 2:**
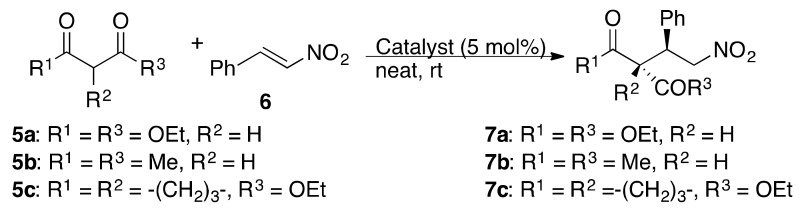
Michael additions to trans-β-nitrostyrene.

The ability of the grafted PIMs (**I**–**VI**) to promote stereoselective transformations was first explored in the nitro-Michael addition and is one of the most widely studied organocatalyzed reactions [46]. The reaction was carried out in neat conditions by stirring a mixture of *trans*-β-nitrostyrene **6** (1 equivalent) with pronucleophiles **5a**–**c** (2 equivalents) and the corresponding catalyst (0.05 equivalents) at room temperature (Scheme 2).

The catalytic efficiency of all the polymers was tested in the reaction of *trans*-β-nitrostyrene with diethyl malonate **5a** (entries 1–6 in Table 1). These data show that the reactions promoted by catalysts derived from PIM-1 (**I**–**III**) are more enantioselective than those catalyzed by polymers derived from PIM-CO-1 (**IV**–**VI**) (compare entries 1–3 versus 4–6). The more acidic 2,4-pentanedione **5b** reacted faster than **5a** with excellent enantioselectivity in the reactions catalyzed by squaramides **II** and **V** (entries 8 and 9) but moderate enantioselection in the presence of thioureas **I** and **VI** (entries 7 and 10). Tertiary nucleophile **5c** also reacted very fast, leading to the addition product **7c** in good diastereoselection but moderate enantioselectivity (entries 11–14).

To test the recyclability of the catalysts, we selected the reaction of diethyl malonate **5a** with *trans*-β-nitrostyrene **6** catalyzed by thiourea **III** (entries 15–19 in Table 1) and the reaction of 2,4-pentanedione **5b** catalyzed by squaramide **II** (entries 20–24 in Table 1) in the described reaction conditions. For each cycle, the catalyst was recovered by filtration, washed, dried under vacuum, and used in the next reaction. The results summarized in Table 1 demonstrate that both the activity and the enantio-discrimination were maintained along six cycles, showing that insignificant deterioration of the catalysts occurred. Additionally, both the IR spectra and analytical data of the recovered catalysts are coincident with those of the starting materials. ^1^H NMR spectra of the final mixtures showed that the transformation was complete for each cycle, and the variations in the isolated chemical yields could be attributed to the efficiency of the flash chromatography in each case. The obtained results are comparable to those obtained with thioureas and squaramides supported on different polymeric materials [43].

**Scheme 3:**
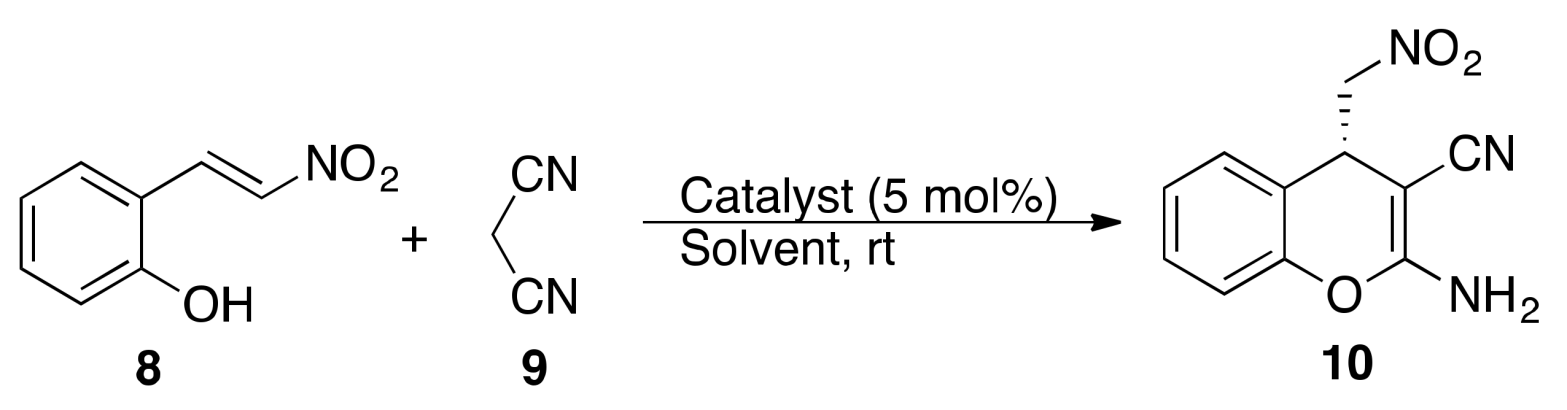
Synthesis of Chromenes by sequential Michael-cyclization process.

The 2-amino-4H-chromenes as biological active compounds [47] lets us consider the synthesis of enantioenriched 2,3,4-trisubstituted 4H-chromenes by using **I**–**VI** as organocatalysts following a cascade process related to others previously described [48]. The reaction of 2-hydroxynitrostyrene (**8**) with malononitrile (**9**) proceeded smoothly in CH_2_Cl_2_, leading to a very good 2-amino-3-cyano-4-nitromethyl-4H-chromene (**10**) yield (Scheme 3) but to an unfortunately moderate enantioselectivity (Table 2).

The stereogenic center was formed in the first step of the process (Michael addition of **9** to **8**), then the cyclization of the addition product, followed by a prototropic displacement in the intermediate lead to the 4H-chromene **10**. As observed for the nitro-Michael reactions described above, the reactions catalyzed by **I**–**III** and derived from PIM-1 were more enantioselective than those promoted by **IV**–**VI** and prepared from PIM-CO-1.

## 4. Conclusions

We have prepared six novel polymeric organocatalysts by grafting two very well-known polymers of intrinsic microporosity (PIM-1 and PIM-CO-100). Interestingly, the polymers conserve their microporous structure after the described modifications.

The organocatalytic activity of these polymers has been tested in the stereoselective nitro-Michael addition and in a cascade nitro-Michael-cyclization process leading to trisubstituted 4H-chromenes. We have also demonstrated that these polymers are recoverable and reusable as organocatalysts. The present study provides a novel application of modified PIMs for catalytic asymmetric transformations, and more work remains to be done to improve their catalytic activity in different stereoselective transformations.

## Figures and Tables

**Table 1:** Nitro-Michael addition of different nucleophiles to *trans*-β-nitrostyrene ^a^.

Entry	5a–c	Catalyst	t (H)	Product (Yield) ^b^	*Dr* ^c^	Er ^d^ (Conf)
1	**5a**	**I**	12	**7a** (71)	-	79:21 (S)
2	**5a**	**II**	12	**7a** (69)	-	81:19 (S)
3	**5a**	**III**	12	**7a** (79)	-	93:7 (R)
4	**5a**	**IV**	6	**7a** (69)	-	68:32 (S)
5	**5a**	**V**	12	**7a** (75)		80:20 (S)
6	**5a**	**VI**	12	**7a** (86)	-	89:11 (R)
7	**5b**	**I**	1	**7b** (68)		74:26 (S)
8	**5b**	**II**	1	**7b** (72)	-	96:4 (S)
9	**5b**	**V**	1	**7b** (75)	-	92:8 (S)
10	**5b**	**VI**	1	**7b** (72)	87:13	71:29 (R)
11	**5c**	**I**	1	**7c** (93)	89:11	79:21 (S,R) ^e^
12	**5c**	**II**	1	**7c** (89)	91:9	81:19 (S,R) ^e^
13	**5c**	**III**	1	**7c** (94)	92:8	65:35 (R,S) ^e^
14	**5c**	**VI**	1	**7c** (87)	88:12	68:32 (R,S) ^e^
15 ^f^	**5a**	**III**	12	**7a** (79)	-	89:11 (R)
16 ^f^	**5a**	**III**	12	**7a** (82)	-	86:14 (R)
17 ^f^	**5a**	**III**	12	**7a** (71)	-	89:11 (R)
18 ^f^	**5a**	**III**	12	**7a** (68)	-	90:10 (R)
19 ^f^	**5a**	**III**	12	**7a** (65)	-	89:11 (R)
20 ^g^	**5b**	**II**	1	**7b** (81)	-	95:5 (S)
21 ^g^	**5b**	**II**	1	**7b** (83)	-	96:4 (S)
22 ^g^	**5b**	**II**	1	**7b** (79)	-	94:6 (S)
23 ^g^	**5b**	**II**	1	**7b** (86)	-	96:4 (S)
24^g^	**5b**	**II**	1	**7b** (86)	-	96:4 (S)

^a^ The reactions were carried out with *trans*-β-nitrostyrene (0.3 mmol), pronucleophiles **5a**–**c** (0.6 mmol, 2.0 equiv.) and the corresponding catalyst (**I**–**VI**) (5 mol %), without solvent. ^b^ Yields correspond to pure isolated compounds. ^c^ Determined by ^1^HNMR in the reaction mixture. ^d^ Determined by HPLC on a chiral column. ^e^ The provided configuration corresponds to the major diastereoisomer. ^f^ Cycles 2–6 for entry 3. ^g^ Cycles 2–6 for entry 8.

**Table 2:** Cascade nitro-Michael-cyclization process.

Entry	Solvent	Catalyst	t (H)	Yield ^a^	*Er*^b^ (Conf)
1	CH_2_Cl_2_	**I**	2	77	80:20 (S)
2	CH_2_Cl_2_	**II**	2	75	67:33 (S)
3	CH_2_Cl_2_	**III**	2	83	75:25 (R)
4	CH_2_Cl_2_	**IV**	2	70	68:32 (S)
5	CH_2_Cl_2_	**V**	2	68	65:35 (S)
6	CH_2_Cl_2_	**VI**	2	76	71:29 (R)

^a^ Yields correspond to pure isolated compounds; ^b^ Determined by HPLC on a chiral column.

## Supplementary Materials

The following are available online at http://www.mdpi.com/2073-4360/11/1/13/s1, Figure S1. ^1^H NMR spectrum of PIM-1 (CDCl_3_, 400 MHz); Figure S2. ^13^C NMR spectrum of PIM-1 (CDCl_3_, 100 MHz); Figure S3. ^1^H NMR spectrum of PIM-CO-10**0** (CDCl_3_, 400 MHz); Figure S4. ^13^C NMR spectrum of PIM-CO-100 (CDCl_3_, 100 MHz); Figure S5. IR (ATR) for thiourea **I**; Figure S6. IR (ATR) for squaramide **II**; Figure S7. IR (ATR) for thiourea **III**; Figure S8. IR (ATR) for thiourea **IV**; Figure S9. IR (ATR) for squaramide **V**; Figure S10. IR (ATR) for thiourea **VI**; Figure S11. HPLC profile for **7a** (racemic); Figure S12. HPLC profile for **7a**. Entry 3, Table 1. 93:7 er; Figure S13. HPLC profile for **7b** (racemic); Figure S14. HPLC profile for **7b**. Entry 8, Table 1. 96:4 er; Figure S15. HPLC profile for **7c** (racemic); Figure S16. HPLC profile for **7c**. Entry 12, Table 1. 91:9 dr; 81:19 er; Figure S17. HPLC profile for **10** (racemic); Figure S18. HPLC profile for **10**. Entry 1, Table 2. 80:20 er.
