# Supplementary material for: Chiral Bifunctional Thioureas and Squaramides Grafted into Old Polymers of Intrinsic Microporosity for Novel Applications
Source: Polymers (Basel). 2018 Dec 21;11(1):13. doi: 10.3390/polym11010013 (PMC6401694; doi:10.3390/polym11010013)
Supplement: Supplementary file 1 [file polymers-11-00013-s001.pdf]

Supplementary Material

Chiral Bifunctional Thioureas and Squaramides Grafted into Old PIMs for Novel Applications.

María Valle, Laura Martín, Alicia Maestro, José M. Andrés,* and Rafael Pedrosa*

Instituto CINQUIMA and Departamento de Química Orgánica, Facultad de Ciencias, Universidad de Valladolid, Paseo de Belén 7, 47011-Valladolid. Spain

E-mail: jmandres@qo.uva.es

E-mail: pedrosa@qo.uva.es

Table of contents

1- Analytical data for enantioselective reaction products **S2**

2. NMR (PIM-1 and PIM-CO-100) **S4**

3. IR (catalysts I-VI) spectra **S6**

4. HPLC chromatograms **S9**

1- Analytical data for enantioselective reaction products

(R)-Diethyl-2-(2-nitro-1-phenylethyl)malonate (7a)[1]

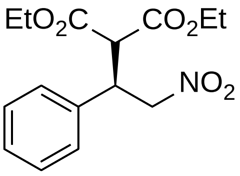

Colorless solid. **[α]$_D^{23}$** = -4.4 (c = 1.0, $CHCl_3$, er 93:7). [Lit.[2] [α]$_D^{30}$ = -6.0 (c = 1.0, $CHCl_3$, 93% ee) for *R* enantiomer]. ^{1}H-NMR (500 MHz, $CDCl_3$): δ 1.05 (t, J = 7.1Hz, 3H), 1.27 (t, J = 7.1Hz, 3H), 3.82 (d, J= 9.3 Hz, 1H), 4.01 (q, J = 7.1 Hz, 2H), 4.13-4.27 (m, 3H), 4.86 (dd, J = 13.1, 9.2 Hz, 1H), 4.92 (dd, J = 13.1, 4.8 Hz, 1H), 7.23-7.33 (m, 5H) ppm. Chiral HPLC analysis**:** Chiralpak AD-H, hexane/2-propanol 80:20, flow rate 1.0 mL/min, λ=220 nm, retention times: t_R=9.9 min (major, R), t_R= 25.4 min (minor, S). (er 93:7).

(S)-3-(2-nitro-1-phenylethyl) pentane-2,4-dione (7b)[2]

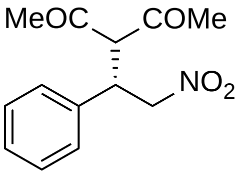

Colorless solid. **[α]$_D^{23}$** = +201.0 (c = 1.0, $CHCl_3$, er 96:4). [Lit.[3] [α]$_D^{23}$ = +196.7 (c = 1.0, $CHCl_3$, er 94:6) for *S* enantiomer]. ^{1}H-NMR (500 MHz, $CDCl_3$): δ 1.92 (s, 3H), 2.27 (s, 3H), 4.20-4.28 (m, 1H), 4.35 (d, J = 10.8 Hz, 1H), 4.58-4.68 (m, 2H), 7.15-7.17 (m, 2H), 7.22-7.35 (m, 3H) ppm. Chiral HPLC analysis**:** Lux Amylose -1, hexane/2-propanol 90:10, flow rate 1.0 mL/min, λ=220 nm, retention times: t_R=12.3 min (major, S), t_R= 18.1 min (minor, R). (er 96:4).

[1] Okino, T.; Hoashi, Y.; Furukawa, T.; Xu, X.; Takemoto, Y. Enantio- and diastereoselective Michael reaction of 1,3-dicarbonyl compounds to nitroolefins catalyzed by a bifunctional thiourea *J. Am. Chem. Soc.* **2005**, 127, 119-125

[2] Rao, K. S.; Trivedi, R.; Kantam, M. L. Ferrocene analogues of hydrogen-bond-donor catalysts: An investigative study on asymmetric Michael addition of 1,3-dicarbonyl compounds to nitroalkenes. *Synlett* **2015**, 26, 221-227.

[3] D.A. Evans, S. Mito, D. Seidel. Scope and mechanism of enantioselective Michael additions of 1,3-dicarbonyl compounds to nitroalkenes catalyzed by Nickel(II)−diamine complexes*J. Am. Chem. So*c. **2007**, *129*, 11583-11592.

(S)-Ethyl 1-((R)-2-nitro-1-phenylethyl)-2-oxocyclopentane-1-carboxylate (7c)[4]

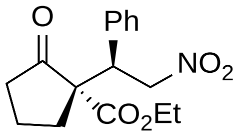

Colorless solid. **[α]$_D^{23}$** = +21.5 (c = 1.0, $CHCl_3$, er 81:19). [Lit.[4] [α]$_D^{25}$ = +30.8 (c = 1.0, $CHCl_3$, 92% ee)]. ^{1}H-NMR (500 MHz, $CDCl_3$): δ 1.27 (t, J = 7.2 Hz, 3H), 1.81-2.06 (m, 4H), 2.30-2.42 (m, 2H), 4.07 (dd, J = 10.9, 3.8 Hz, 1H), 4.21 (m, 2H), 5.01 (dd, J = 13.6, 11.0 Hz, 1H), 5.17 (dd, J = 13.6, 3.8 Hz, 1H), 7.25-7.32 (m, 5H) ppm. Chiral HPLC analysis**:** Chiralcel OD, hexane/2-propanol 80:20, flow rate 1.0 mL/min, λ=220 nm, retention times: t_R (major diastereoisomer) = 7.6 min (major; *S*, *R*), 10.0 min (minor; *R*, *S*). (dr 91:9; er 81:19).

(*S*)-2-Amino-4-(nitromethyl)-4*H*-chromene-3-carbonitrile (10).[5]

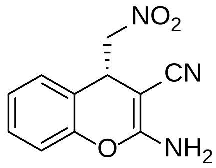

Colorless solid. **[α]$_D^{23}$** = +10.3 (c = 1.0, $CHCl_3$, er 80:20). [Lit.[5][α]$_D^{23}$ = +24.0 (c = 1.0, $CHCl_3$, 84% ee for 4*S* enantiomer]. **^{1}H-NMR** (DMSO-d_6, 400 MHz): d 4.31 (t, J=5.2 Hz, 1H, C<u>H</u>), 4.66 (dd, J= 12.4, 5.2 Hz, 1H, C<u>H</u>$_2$), 4.79 (dd, J = 12.4, 5.2 Hz, 1H, C<u>H</u>$_2$), 7.03 (d, J = 8.0 Hz, 1H, <u>H</u>ar), 7.17 (d, J = 9.2 Hz, 1H, <u>H</u>ar), 7.18 (br s, 2H, N<u>H</u>$_2$), 7.32 (t, J= 8.4 Hz, 2H, <u>H</u>ar). **HPLC**: (Chiralpak AD-H column, *n*-hexane/*iso*-propanol = 80:20, 1 mL/min, λ = 254 nm) t_R = 10.7 min (major, *S*), t_R = 12.1 (minor, *R*). (er 80:20).

[4] Manzano, R.; Andrés, J. M.; Muruzábal, M. D.; Pedrosa, R. Stereocontrolled construction of quaternary stereocenters by inter- and intramolecular nitro-Michael additions catalyzed by bifunctional thioureas. *Adv. Synth. Catal.* **2010**, 352, 3364-3372

[5] K. Hu, Y. Wang, Z. Zhou, C. Tang. Novel thiophosphonodiamides as efficient hydrogen bond donor catalysts in tandem Michael addition–cyclization of malononitrile and 2-(E)-2-nitrovinylphenols. *Tetrahedron* **2014**, *70*, 181-185

2. NMR (PIM-1, PIM-CO-100)

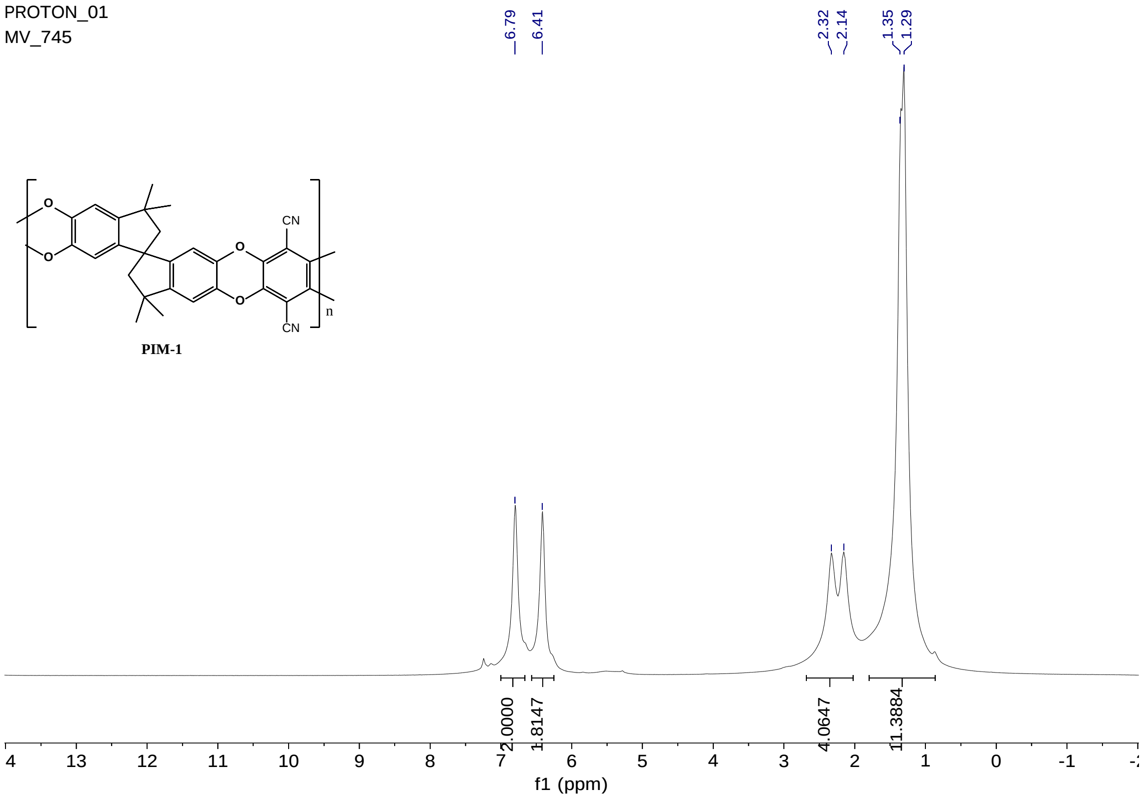

Figure S1. ^{1}H NMR spectrum of **PIM-1** ($CDCl_3$, 400 MHz).

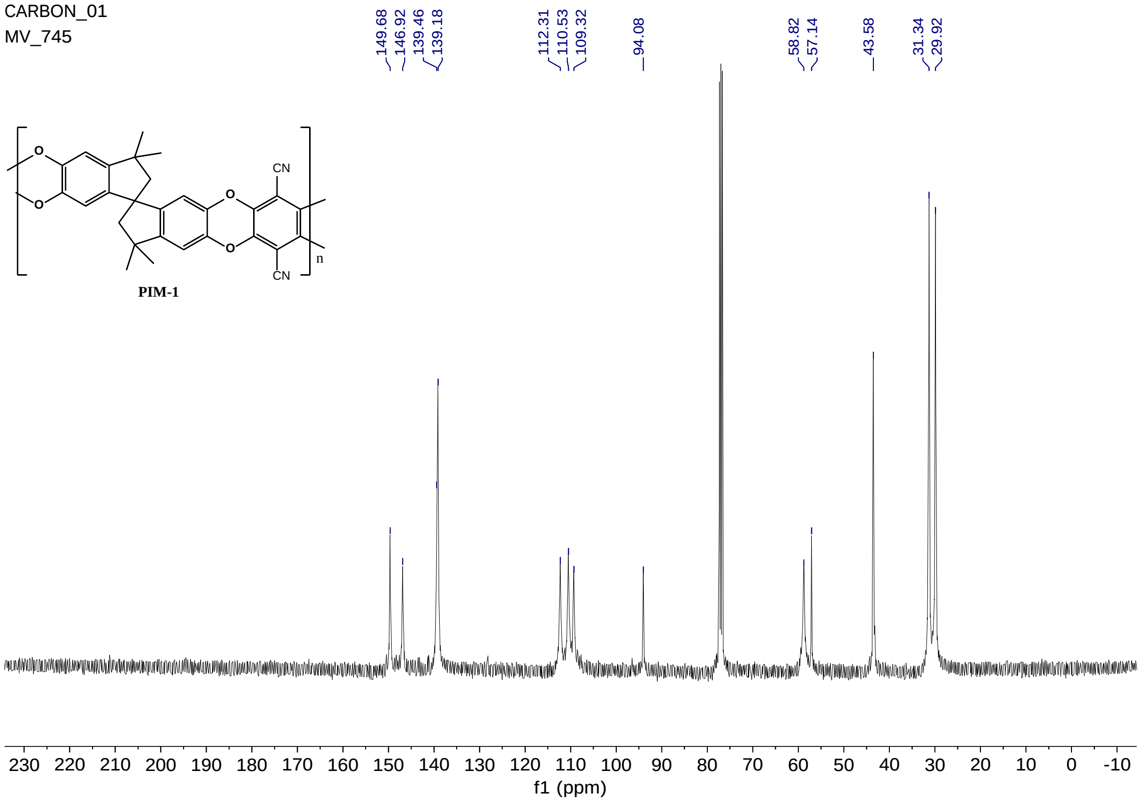

Figure S2. ^{13}C NMR spectrum of **PIM-1** ($CDCl_3$, 100 MHz).

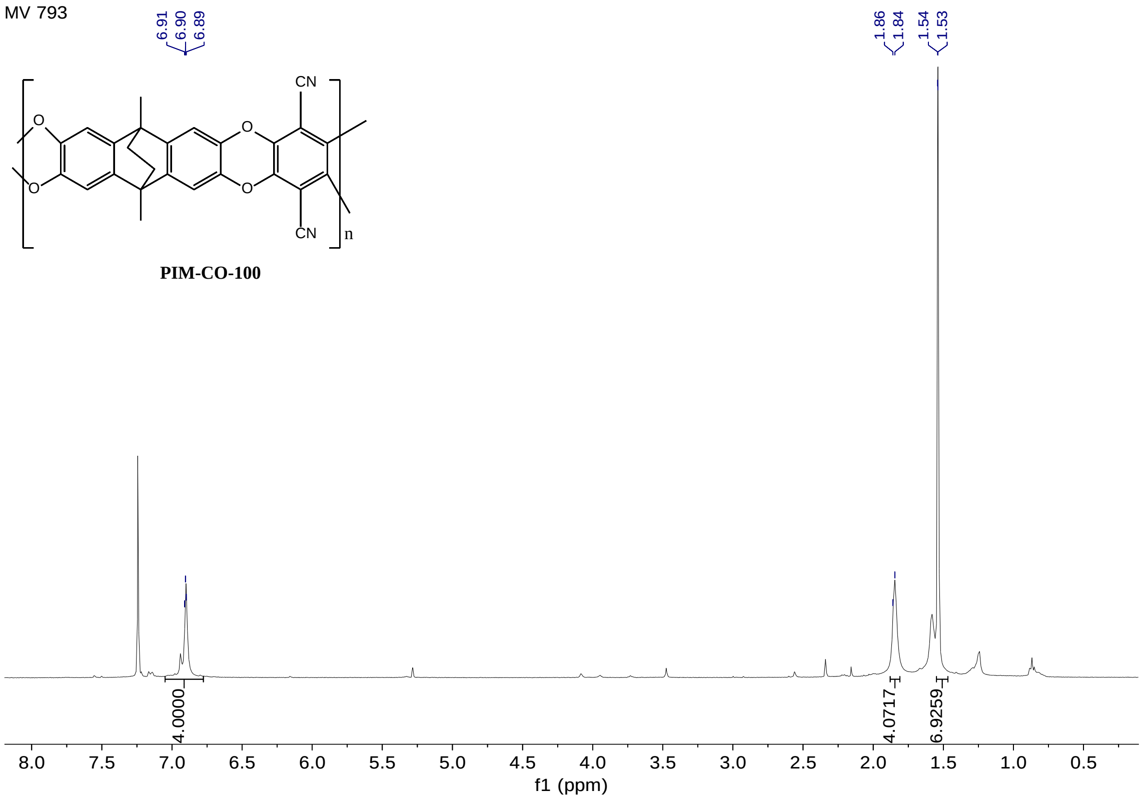

Figure S3. ^{1}H NMR spectrum of **PIM-CO-100** ($CDCl_3$, 400 MHz).

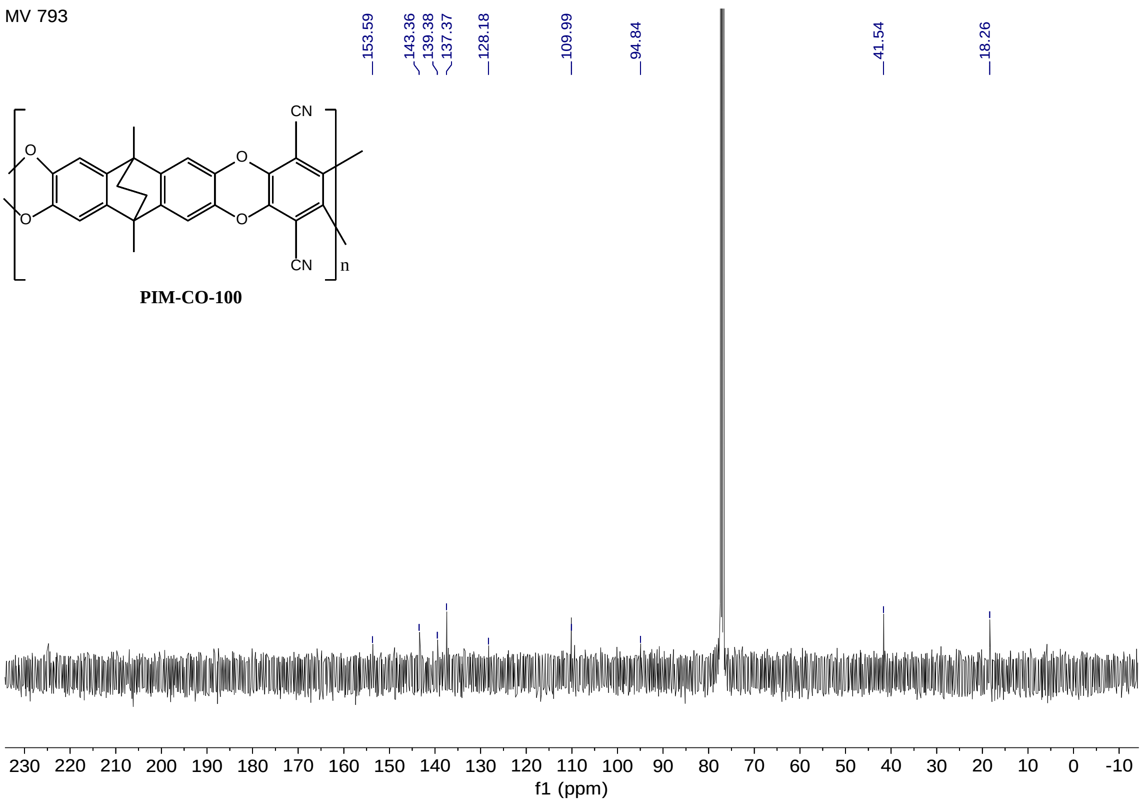

Figure S4. ^{13}C NMR spectrum of **PIM-CO-100** ($CDCl_3$, 100 MHz).

3. IR (catalysts I-VI) spectra

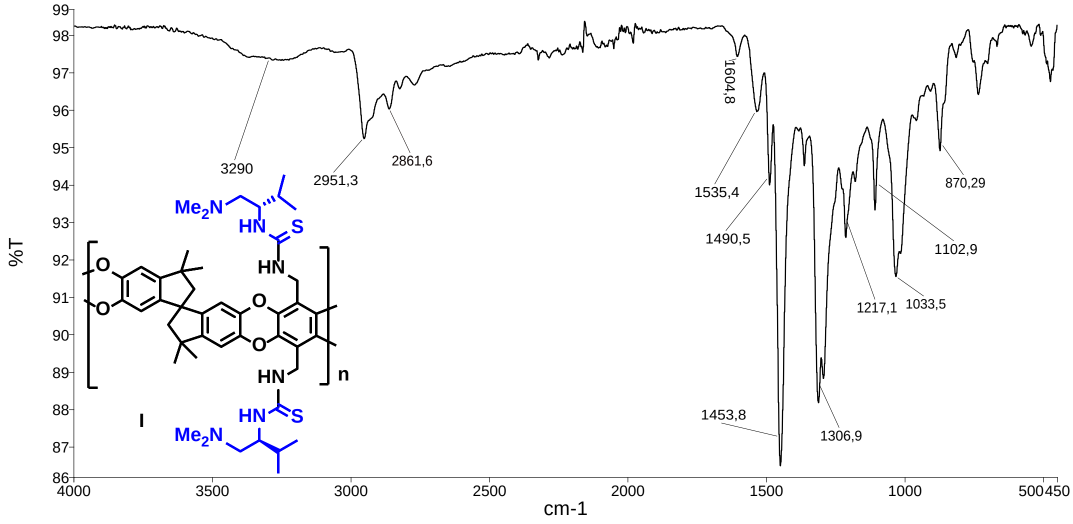

Figure S5. IR (ATR) for thiourea **I**.

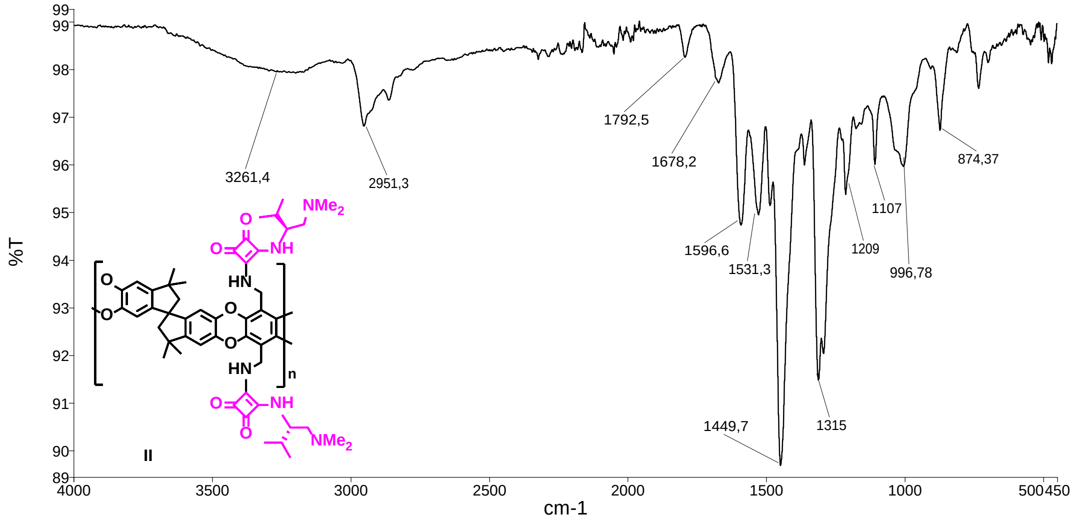

Figure S6. IR (ATR) for squaramide **II**.

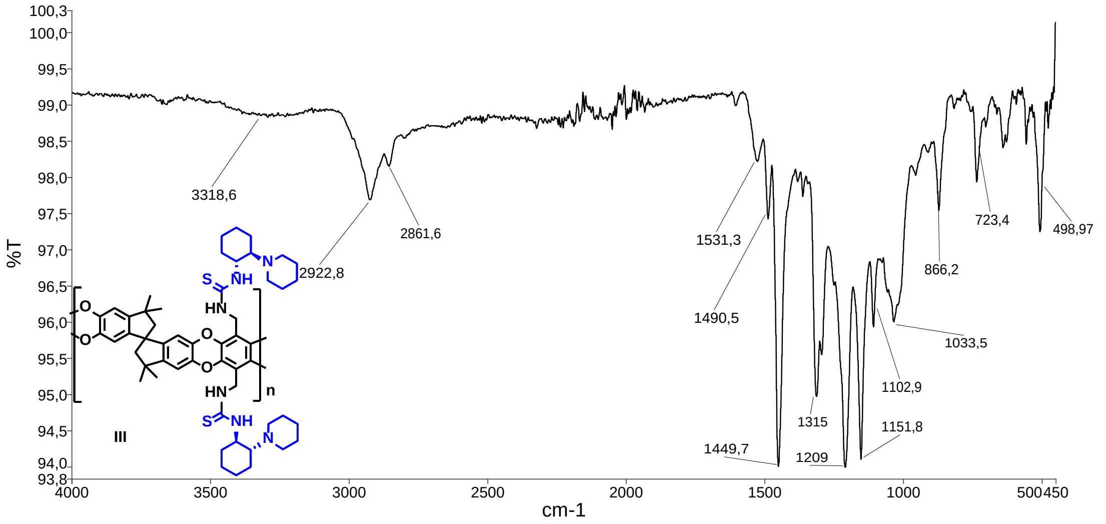

Figure S7. IR (ATR) for thiourea **III**.

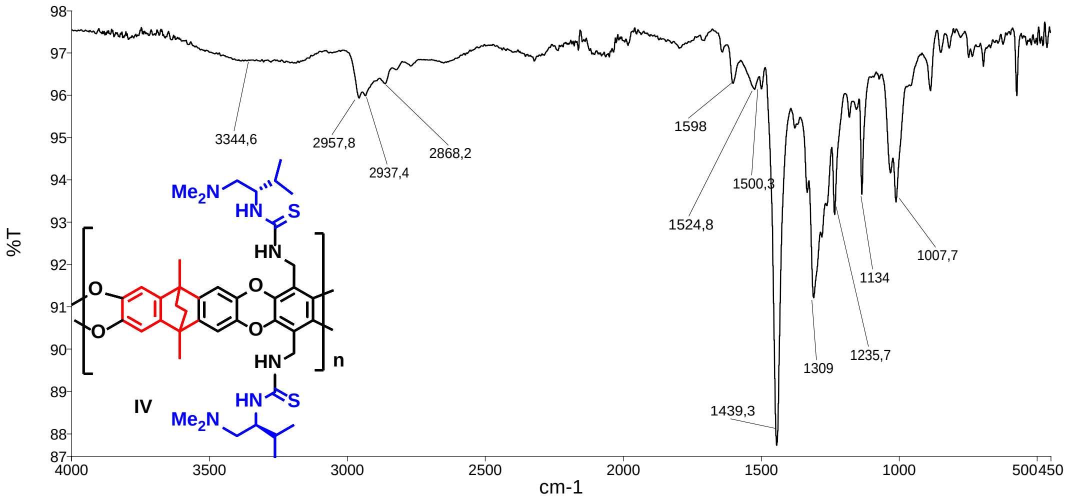

Figure S8. IR (ATR) for thiourea **IV**.

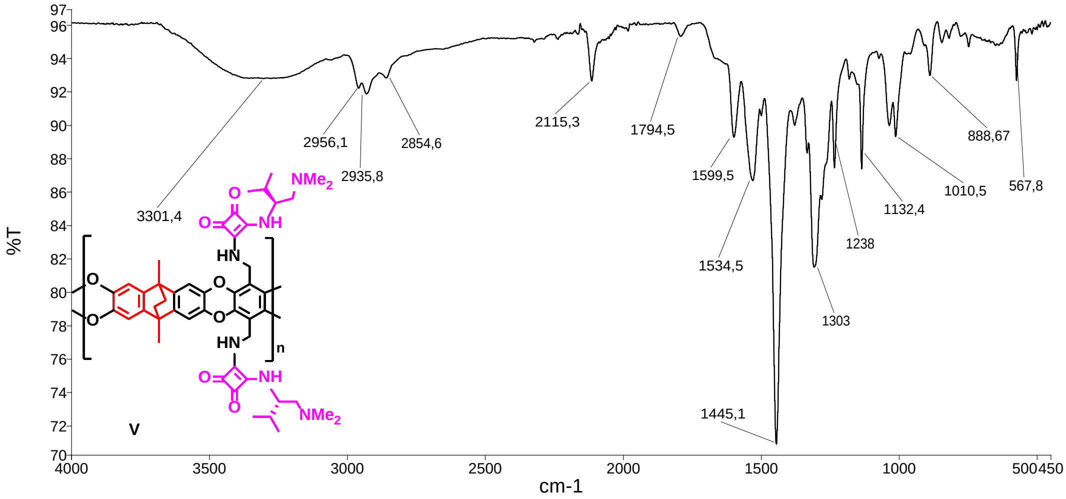

Figure S9. IR (ATR) for squaramide **V**.

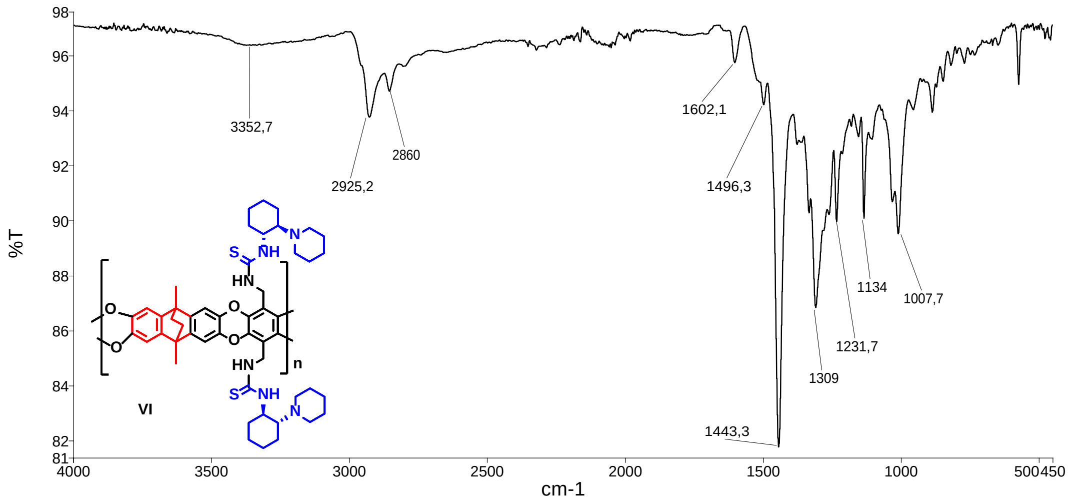

Figure S10. IR (ATR) for thiourea **VI**.

4. HPLC chromatograms

(R)-Diethyl-2-(2-nitro-1-phenylethyl)malonate (7a).

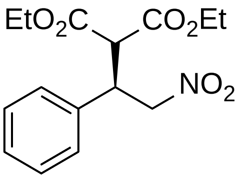

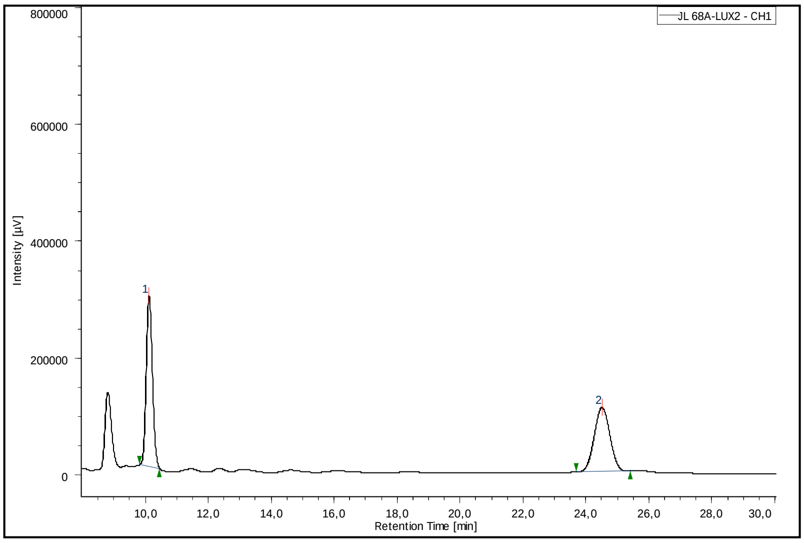

Peak Name	t_R	Area	Height	Area%	Height%	Symmetry
1	**10,125**	4030123	291888	**51,725**	72,735	1,081
2	**24,483**	3761379	109417	**48,275**	27,265	1,023

Figure S11. HPLC profile for **7a** (racemic).

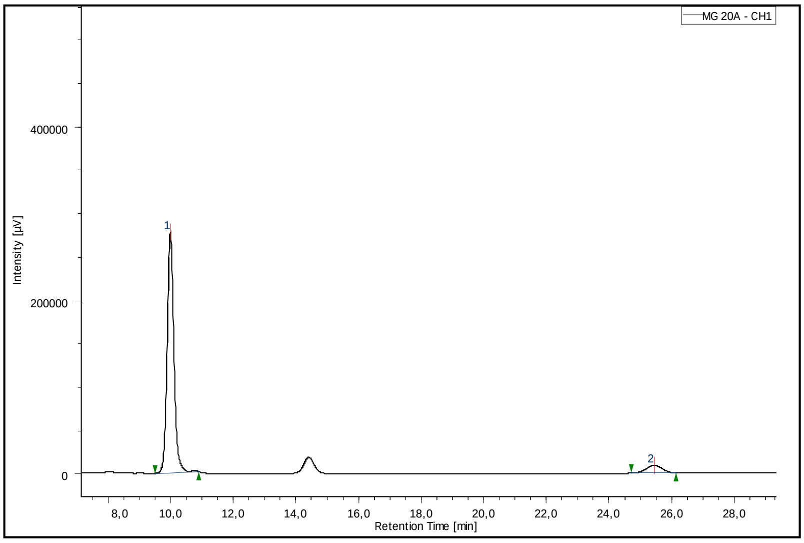

Peak Name	t_R	Area	Height	Area%	Height%	Symmetry
1	**9,983**	4170436	276207	**92,925**	96,886	1,137
2	**25,433**	317526	8877	**7,075**	3,114	0,971

Figure S12. HPLC profile for **7a**. Entry 3, table 1. 93:7 er.

(S)-3-(2-nitro-1-phenylethyl) pentane-2,4-dione (7b).

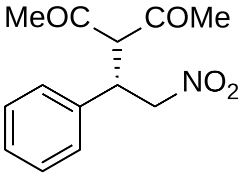

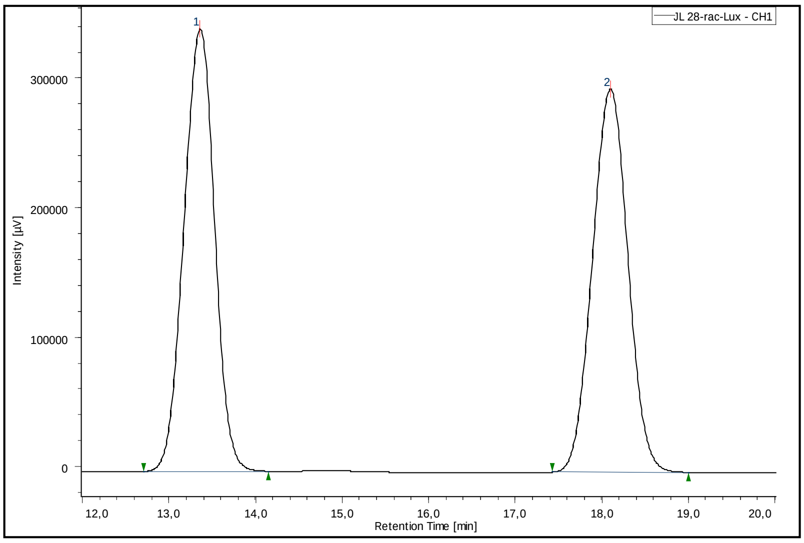

Peak Name	t_R	Area	Height	Area%	Height%	Symmetry
1	**13,358**	8391541	340752	**49,931**	53,568	0,983
2	**18,092**	8414827	295358	**50,069**	46,432	1,024

Figure S13. HPLC profile for **7b** (racemic).

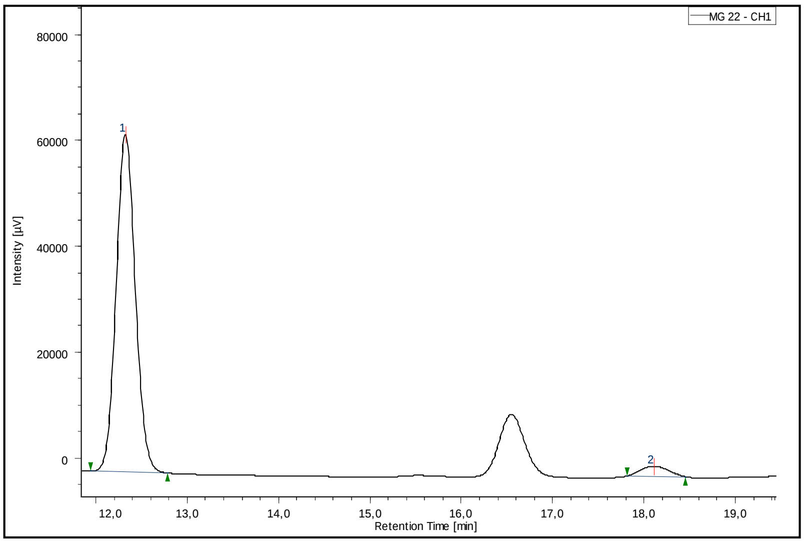

Peak Name	t_R	Area	Height	Area%	Height%	Symmetry
1	**12,325**	926480	63637	**95,947**	97,112	1,074
2	**18,100**	39136	1892	**4,053**	2,888	1,074

Figure S14. HPLC profile for **7b**. Entry 8, table 1. 96:4 er.

(S)-Ethyl 1-((R)-2-nitro-1-phenylethyl)-2-oxocyclopentane-1-carboxylate (7c).

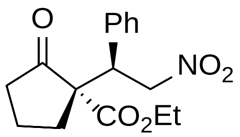

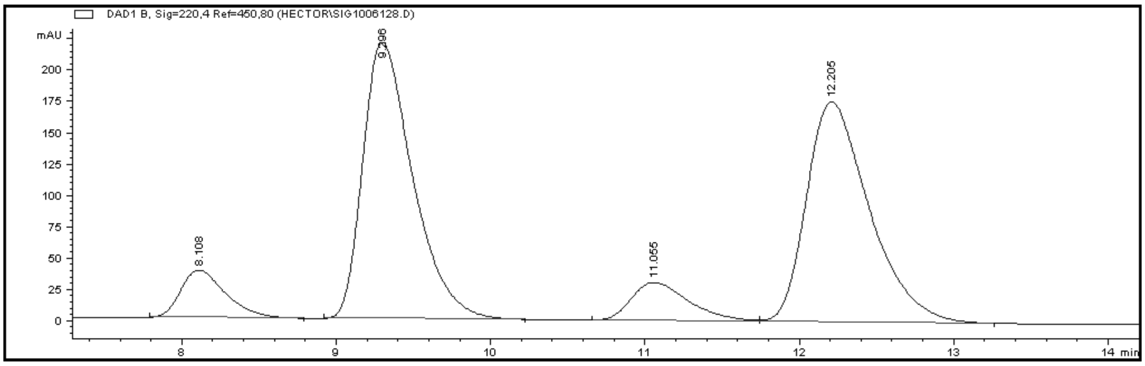

Peak Name	t_R	Area	Height	Width	Area%	Symmetry
1	8,108	772,2	37,9	0,3146	6,702	0,642
**2**	**9,296**	4974,3	220,7	0,3462	**43,173**	0,604
3	11,055	790,4	30,6	0,3652	6,860	0,613
**4**	**12,205**	4984,9	175,9	0,433	**43,265**	0,625

Figure S15. HPLC profile for **7c** (racemic).

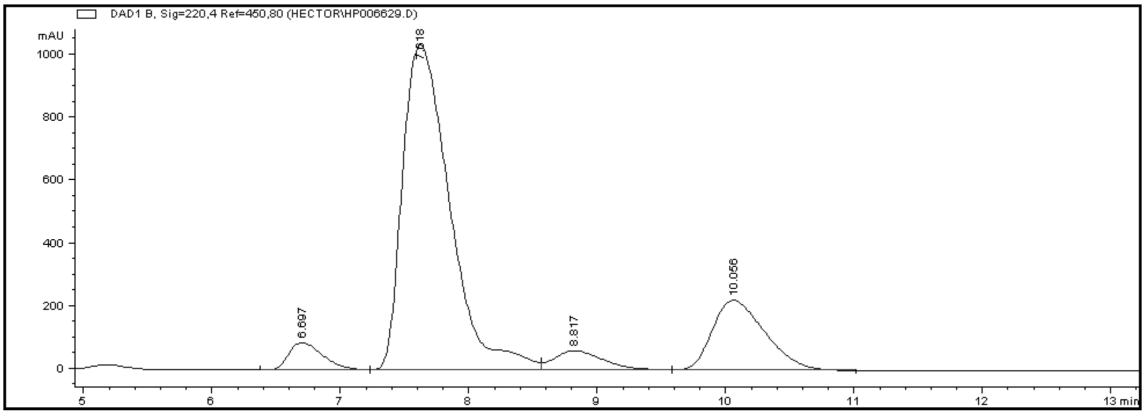

Peak Name	t_R	Area	Height	Width	Area%	Symmetry
1	6,697	1616,7	86,9	0,2874	4,468	0,602
**2**	**7,618**	26532,6	1035,2	0,4014	**73,325**	0,557
3	8,817	1692,5	62,6	0,3955	4,677	0,633
**4**	**10,056**	6343,2	222,8	0,4313	**17,530**	0,638

Figure S16. HPLC profile for **7c**. Entry 12, table 1. 91:9 dr; 81:19 er.

(*S*)-2-Amino-4-(nitromethyl)-4H-chromene-3-carbonitrile (10).

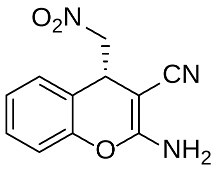

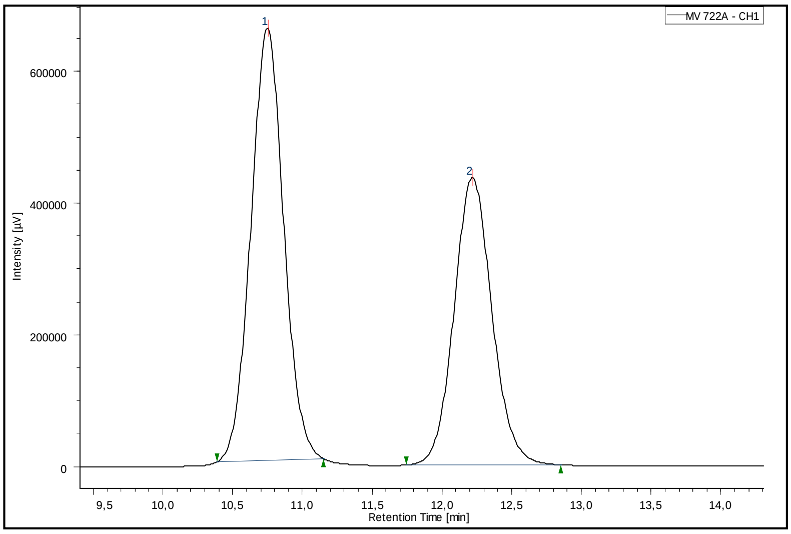

Peak Name	t_R	Area	Height	Area%	Height%	Symmetry
**1**	**10,750**	10728205	654183	**50,584**	60,043	1,026
**2**	**12,217**	10480487	435334	**49,416**	39,957	1,080

Figure S17. HPLC profile for **10** (racemic).

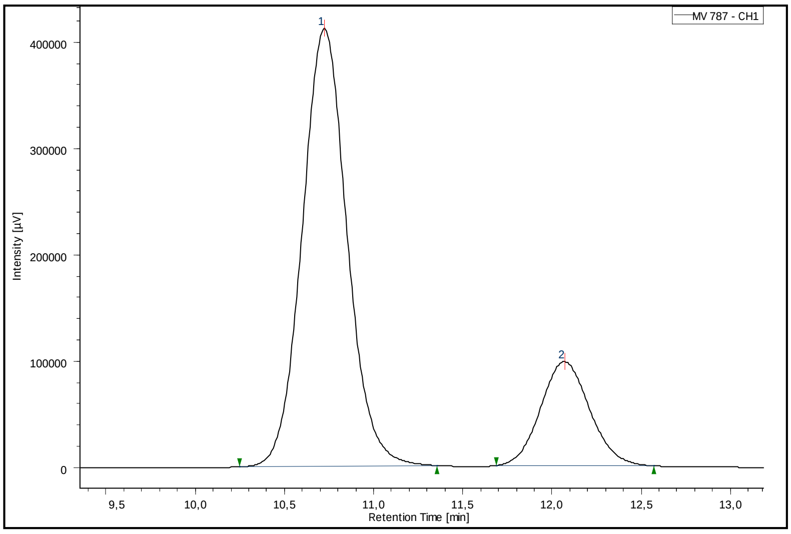

Peak Name	t_R	Area	Height	Area%	Height%	Symmetry
**1**	**10,725**	7239406	410930	**79,511**	80,807	1,060
**2**	**12,067**	1865474	97605	**20,489**	19,193	1,084

Figure S18. HPLC profile for **10**. Entry 1, table 2. 80:20 er.
